# German Poison Eaters

**Published:** 1854-11

**Authors:** 


*German Poison Eaters.*—Dr. Tschudi has published, in the *Wiener Medizinische Wochenschrift* two letters, the translation of which is to be found in the *Journal de Médecine de Bruxelles,* containing some curious details relative to a class of people who are habitual arsenic-eaters.

In some countries of lower Austria and of Styria, especially in the mountains which separate these parts from Hungary, there exists among the peasantry the singular habit of eating arsenic. They purchase it under the name of hedri (*hedri, hedrich, hatterrauch*) from wandering herbalists, or pedlars, who, on their part, obtain it from workers in Hungarian glass, from veterinary surgeons, from charlatans.

These poison-eaters (*toxicophagi*) have a double aim ; first, they wish to give themselves, by this dangerous habit, a fresh and healthy appearance, and a certain degree of embonpoint. Many of the peasant girls, and even the men, have recourse to this expedient from coquetry and a desire to please ; and it is remarkable what success they attain, for the young toxicophagi are distinguisbed by the freshness of their complexion and by the aspect of flourishing health. The following is one of many instances. A girl who attended cows, in good health, but pale and thin, was employed at a farm in the parish of H——. Having a lover, whom she wished to attract yet more by her personal charms, she had recourse to the usual method, and took arsenic several times a week. The desired result was soon obtained ; and after some months, she became fat, chubby-cheeked, and, in short, quite to Celadon's taste. To carry the effect further, she increased the dose, and fell a victim to her "coquetterie ;" she died poisoned. The number of deaths from the abuse of arsenic is by no means inconsiderable, especially among the young people. Every ecclesiastic in those parts can speak of several victims, and Dr. Tschudi states that his researches among the clergy were very interesting. He learned that so careful were the victims of this practice to conceal what they had done, that the secret was often revealed only on the death-bed.

The second advantage gained by the toxicophagi is, that they become more "volatile," more free in respiration, and able to ascend high mountains with ease. Upon every long excursion into the mountains they take a little bit of arsenic, which they let dissolve in the mouth. The effect is surprising. They ascend without difficulty heights which would have been almost insurmountable without this practice. The author adds, that upon this experience, he has advantageously administered Fowler's solution in cases of asthma.

The toxicophagi commence with a bit of arsenic the size of a lentil-seed, or about half-a-grain. They keep to this dose, which they swallow several times a-week, morning and evening, for a long period, to become accustomed to it. Then they increase the quantity insensibly, but with precaution, until the desired effect is produced. A countryman, named R——, of the commune Ag——, a sexagenarian, and in excellent health, was in the habit of daily taking four grains. He had followed the habit forty years, and had transmitted it to his son. There was no trace of arsenical cachexia in this individual, no symptoms of chronic poisoning. It is to be remarked, however, that, when the practice is dropped, emaciation generally ensues from some cause, either from the withdrawal of the stimulus, or from accidental or acquired disease. The custom does not diminish the sexual passion, as is the case with the opiophagi of the East, or with those who use the betel in India and Polynesia. On the contrary, the feeling becomes more strong.

It may be as well to bring to mind a general use of arsenic in Vienna, among the stablemen and coachmen of the great houses. They mix a good pinch of the powder with corn, put a piece the size of a pea in a linen bag, and attach it to the bit of the horse. The saliva dissolves the poison. This produces a bright aspect of the skin, roundness and elegance of form, and foam at the mouth. The coachmen of the hills adopt the same practice before commencing a laborious journey; and horse-dealers carry with them small balls of arsenic, to be given to those animals which are being led to the market. Should a horse thus treated pass into the hands of a master who does not employ arsenic, he gets thin, loses his freshness, becomes dull, and in spite of abundant food does not acquire his former sleekness.—*London Chemist, from Gaz. des Hôpitaux.*

